# *Plasmodium falciparum* Genetic Diversity in Panamá Based on *glurp*, *msp*-1 and *msp*-2 Genes: Implications for Malaria Elimination in Mesoamerica

**DOI:** 10.3390/life10120319

**Published:** 2020-11-28

**Authors:** Ana María Santamaría, Vanessa Vásquez, Chystrie Rigg, Dianik Moreno, Luis Romero, Carlos Justo, Luis Fernando Chaves, Azael Saldaña, José E. Calzada

**Affiliations:** 1 Departamento de Investigación en Parasitología, Instituto Conmemorativo Gorgas de Estudios de la Salud, Panamá 0816-02593, Republic of Panama; asantamaria@gorgas.gob.pa (A.M.S.); vvasquez@gorgas.gob.pa (V.V.); chrigg@gorgas.gob.pa (C.R.); asaldana@gorgas.gob.pa (A.S.); 2 Facultades de Ciencias Naturales Exactas y Ciencias de la Salud, Universidad de Panamá, Panamá 4 3366, Republic of Panama; 3 Laboratorio Central de Referencia en Salud Publica, Instituto Conmemorativo Gorgas de Estudios de la Salud, Panamá 0816-02593, Republic of Panama; dmoreno@gorgas.gob.pa (D.M.); lromero@gorgas.gob.pa (L.R.); cjusto@gorgas.gob.pa (C.J.); 4 Instituto Costarricense de Investigación y Enseñanza en Nutrición y Salud (INCIENSA), Tres Ríos 4-2250, Cartago 1, Costa Rica; lfchavs@gmail.com

**Keywords:** *Plasmodium falciparum*, genotype, molecular epidemiology, Panamá, Mesoamerica

## Abstract

Panamá, together with all the nations in Mesoamerica, has committed to eliminate malaria from the region by 2020. As these countries approach malaria elimination and local transmission decreases, an active molecular surveillance to identify genotypes circulating along the border areas is particularly needed to accurately infer infection origin, drug resistance and disease propagation patterns in the region. This study evaluated the genetic diversity and allele frequencies of *msp*-1, *msp*-2 and *glurp* genes using different molecular analyses (nested PCR, PCR-restriction fragment length polymorphism (RFLP) and sequencing) from 106 autochthonous and imported *P. falciparum* isolates collected from different endemic areas in Panamá between 2003 and 2019. We also explored if *P. falciparum* genotypes assessed with these molecular markers were associated with relevant malaria epidemiological parameters using a multiple correspondence analysis. A strong association of certain local haplotypes with their geographic distribution in endemic areas, but also with parasite load and presence of gametocytes, was evidenced. Few multiclonal infections and low genetic diversity among locally transmitted *P. falciparum* samples were detected, consequent with the low transmission intensity of this parasite in Panamá, a pattern likely to be extended across Mesoamerica. In addition, several imported cases were genetically dissimilar to local infections and representative of more diverse extra-continental lineages.

## 1. Introduction

Malaria continues to be a major, though neglected, public health challenge for health authorities in Panamá. Despite regional efforts and a national commitment to eliminate locally transmitted malaria cases by 2020 and achieve full WHO certification by 2025 [1], recent studies have evidenced that this goal will be difficult to achieve [2,3,4,5,6,7]. Indeed, the country has not been able to reduce transmission particularly in indigenous endemic regions, registering in the past decade more than 500 annual cases, with a peak of 1400 cases in 2019 [7].

Although *Plasmodium vivax* has been responsible for most malaria cases in Panamá (~94% during the last 15 years) [6], *P. falciparum* resurgence has been a constant threat for malaria control and elimination. For instance, during a major malaria epidemic observed between 2002 and 2003, autochthonous *P. falciparum* transmission was reestablished east of the Panamá Canal, with several fatal cases—a situation not observed in the county since 1970 [7]. Parasites circulating at that time presented mutations associated with resistance to chloroquine (CQ) and partial resistance to sulfadoxine–pyrimethamine (SP) [8,9,10]. This situation prompted a change in the national malaria drug policy in Panamá from CQ to SP, and shortly after to mefloquine, as first-line treatments for uncomplicated *P. falciparum* infections [7,9,11]. Intensification of malaria control activities coupled with the change in drug policy was highly effective to control the 2002–2003 *P. falciparum* outbreak [3,5,6]. Nowadays, following international guidelines, artemisinin-based combination therapy (ACT) is used to treat *P. falciparum* infections in Panamá [11,12]. More recently, since 2015 *P. falciparum* transmission has again re-emerged in eastern regions of the country where malaria transmission had been previously interrupted—An event that has been related to migratory events across the Panamá–Colombia border [10,13]. Due to its strategic geographical position, as a bridge between two oceans and connecting North and South America, Panamá has historically served as an attractive intercontinental passageway for migration. In fact, during the last decade, a significant and increasing number of *P. falciparum* cases have been imported from endemic countries of different continents, most imported cases coming from South America [7].

The National Malaria Elimination Programme (NMEP) in Panamá is now implementing its strategic objectives as part of ongoing malaria elimination efforts [1]. In this context, knowledge of the genetic variations and understanding the population structure of *P. falciparum* parasites circulating in the country can provide to the NMEP new biological insights on the dynamics of parasite transmission, resistance to malaria drugs, infection origin and connectivity between infections [10]. Therefore, a sustained molecular surveillance of *Plasmodium* spp. is key to optimize and effectively implement malaria elimination strategies in Panamá—a country with permeable boundaries in Mesoamerica and with a growing number of migrants entering from different malaria extra-continental endemic regions [7].

In this regard, few studies have explored the genetic diversity of *P. falciparum* parasites circulating in Panamá [8,9,10]. Overall, results from these studies point to a clonal origin of infection and to a CQ resistance pattern of *P. falciparum* circulating in the studied areas of the country [8,10]. This epidemiological scenario is different from the one observed throughout the rest of Mesoamerica where autochthonous *P. falciparum* parasites are currently susceptible to CQ [14]. Results from these previous studies, however, are limited by the low number of samples, the restricted geographical range from where the samples were obtained and particularly the short time span of sample collection, mostly related to the 2012–2014 *P. falciparum* epidemic. In this line, and to provide additional information to optimize the NMEP strategies, the present study evaluated the genetic diversity and allele frequencies of merozoite surface proteins 1 and 2 (*msp*-1 and *msp*-2) and the glutamate-rich protein (*glurp*) genes from autochthonous and imported *P. falciparum* human isolates collected in different endemic areas from Panamá between 2003 and 2019. We also explored if *P. falciparum* genotypes assessed with these molecular markers were associated with relevant malaria epidemiological parameters.

## 2. Materials and Methods

### 2.1. Study Sites and Sample Collection

**Figure 1:**
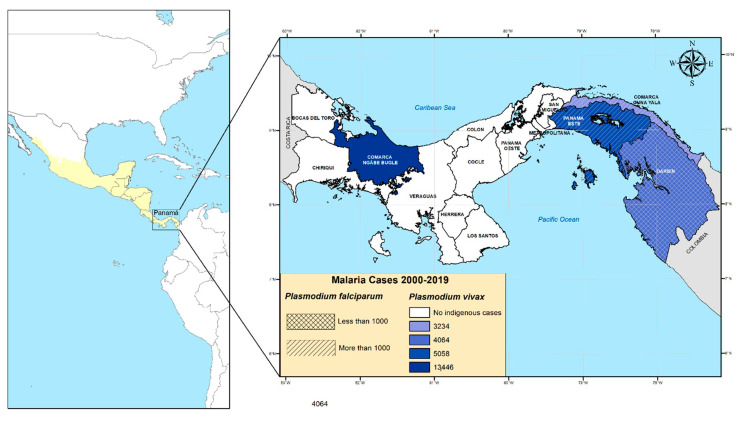
Map of America showing Panamá within the Mesoamerican region (colored in yellow); to the right is a map of Panamá showing malaria health regions with active transmission based on the cumulative number of *Plasmodium falciparum* and *Plasmodium vivax* cases between 2000 and 2019.

Panamá is in Mesoamerica, bordering the Caribbean Sea and the North Pacific Ocean, between Colombia and Costa Rica (Figure 1). The country has slightly over four million inhabitants and occupies the southeastern end of the narrow isthmus forming the land bridge connecting North and South America. It has a tropical climate, with relatively high temperatures throughout the year, ranging from 26 to 32 °C, and two marked seasons: dry season: January–May; and rainy season: May–December.

During the past decade, malaria cases have shown a marked seasonality with more cases occurring during the rainy months [5,7,13]. Moreover, during this period, a disproportionate number of malaria cases (~90%) have been concentrated in the so-called comarcas: semi-autonomous areas inhabited by diverse indigenous groups that occupy 22% of the national territory and together represent around 12% of the total Panamanian population [7,15]. In relation to the species distribution in these malaria hotspots, it is noteworthy that *P. vivax* infections have been widespread throughout the country, whereas during the past decades, *P. falciparum* autochthonous transmission has only been observed in communities located on the eastern side of the country near the Colombian border (Figure 1), suggesting an imported initial origin of *P. falciparum* local transmission.

Malaria surveillance in Panamá is carried out by a joint and coordinated effort between the Departamento de Control de Vectores from the Ministry of Health (DCV-MoH) and Instituto Conmemorativo Gorgas de Estudios de la Salud (ICGES), the national reference diagnostic laboratory [11]. As stated by the national guidelines for malaria control, DCV-MoH performs the search of suspected cases, blood sample collection, treatment and documentation of all cases detected via active and passive search, while ICGES is responsible for supervising microscopic diagnosis and performing molecular diagnosis, as well as *Plasmodium* genotyping of special cases. Routinely, all positive slides, and 10% of the negative slides, are confirmed at the Public Health Central Reference Laboratory by ICGES [11].

### 2.2. Plasmodium Diagnosis by Microscopy and PCR

Capillary blood samples analyzed in this study were obtained by fingerprick from autochthonous and imported suspected malaria cases between 2002 and 2019. Blood samples were used to prepare thick blood smears and spotted onto filter paper for molecular analysis. Giemsa-stained smears were examined for *Plasmodium* spp. and parasite density following the national guidelines for malaria control of the Ministry of Health of Panamá [11]. The presence of *Plasmodium* spp gametocytes in blood smears was also recorded. Parasitemia was classified as low, moderate or high according to the number of parasites observed per microscopic field [16]. To confirm *P. falciparum* positivity, we used a nested PCR that amplifies the small sub-unit ribosomal ribonucleic acid (ssrRNA) genes, following a slightly modified methodology from the protocol of Snounou et al. [17]. Specifically, in the second PCR reaction, only specific primers for *P. falciparum* and *P. vivax* were included, as other *Plasmodium* spp. (*P. ovale*, *P. malaria* and *P. knowlesi*) causing malaria in humans have not been reported in Panamá in more than four decades [7]. All mixture and amplification conditions were previously described [17].

### 2.3. Plasmodium falciparum Genotyping

Genomic DNA was isolated from dried blood spots using a commercial kit (QIAamp DNA mini kit, Qiagen, Hilden, Germany) following the protocol suggested by the manufacturer for dried spot blood. Genetic diversity of confirmed *P. falciparum* samples was assessed by genotyping the conventional molecular markers *glurp*, *msp*-1 and *msp*-2 [18]. For *glurp* and *msp*-1, a nested PCR approach was performed with specific primers and methods as previously described [18]. All amplification reactions were carried out in a total volume of 25 µl using the premixed solution PCR Nucleotide Mix (Promega, Madison, WI, USA). Two microliters of the primary PCR product was used as template for the nested reactions using specific primers for region 2 of *msp*-1 and the RII block of *glurp* [19]. Amplification products were observed on 2% agarose gels containing ethidium bromide, and their sizes were estimated by visual inspection using a 100 bp DNA ladder as the molecular size marker (Promega, Madison, WI, USA). Genotypes of *glurp* and *msp*-1 genes were assigned according to the molecular weights of the amplified products. The detection of a single PCR fragment for each locus indicated an infection with one parasite genotype and was classified as a monoclonal infection. The presence of two or more different size bands in the same sample was considered as a polyclonal infection. To minimize bias, gels were analyzed independently by at least two researchers and gels were run with an appropriate molecular size marker for comparison.

For *msp*-2, a previously described Polymerase Chain Reaction–Restriction Fragment Length Polymorphism (PCR-RFLP) approach was used to evaluate its allelic diversity (FC-27 and 3D7) [20]. Briefly, the nested PCR products were subjected to site-specific restriction enzyme digestion with Hinf I (New England Biolabs, Ipswich, MA, USA) and analyzed on 2% agarose gels. Alleles were assigned based on the restriction banding patterns observed [19]. Specifically, this strategy permits an initial grouping in two distinct families, the FC27 type and the 3D7 type, which are characterized by the presence of conserved fragments. Within each family, a diversity of alleles can be distinguished by the size of variable fragments [20,21,22]. Haplotypes were assigned in sequential numerical orders by combining the genetic variants detected in the three loci (*glurp*, *msp*-1 and *msp*-2) analyzed in this study.

A phylogenetic analysis based on the *msp*-2 partial sequence was further performed by the maximum likelihood method. For this purpose, *msp*-2 nested PCR products from the block 3 region amplified as previously described [20] were electrophoresed on a 1.5% agarose gel, purified using Qiagen DNA purification kit (Qiagen, Hilden, Germany) and then directly sequenced in both directions using an ABI Prism 3500 XL130 sequencer (Applied Biosystems, Foster City, CA, USA). The sequences were edited and aligned with Sequencher 4.1.4 (Gene Codes Corporation, Ann Arbor, MI, USA) and Molecular Evolutionary Genetics Analysis (MEGA) 7.0 software (Pennsylvania State University, Center, PA, USA). Phylogenetic trees were constructed using MEGA 7.0 by the maximum likelihood method with 1000 bootstrap replicates. Nucleotide sequences of msp-2 from this study were submitted to GenBank.

### 2.4. Data and Statistical Analysis

The frequency of *glurp*, *msp*-1 and *msp*-2 alleles was calculated as the proportion of the allele detected for each allelic family out of the total alleles detected. The frequency of polyclonal infection was calculated based on the number of samples with more than one amplified fragment out of the total samples. Single infections were those with only one allele per locus at all the genotyped loci. Multiplicity of infection (MOI) was defined as the largest number of alleles at any locus.

To analyze the pattern of association between haplotypes, built by combining genetic variants of the loci under study with malaria epidemiological parameters, a multiple correspondence analysis (MCA) was fitted with the command mca from the library MASS [23] using the statistical software R version 3.6.1 (R Core Team, Vienna, Austria). Briefly, MCA is a tool that allows studying the association between categorical variables and it is estimated by performing a singular value decomposition (SVD) on a table with counts for categories from the different studied variables that co-occur across the studied subjects [24]. Then, the original data can be projected into the two vectors associated with the largest singular values from the SVD. The resulting values are “coordinates”, including centroids for all levels from the different categorical variables considered in the analysis. The centroids are plotted in two dimensions, thus allowing the evaluation of associations between categories from different variables [23,24]. Associations are stronger as categories from different variables are together but farther apart from the origin (coordinates 0,0 in the 2-D plot) which is the geometric point where random associations are expected to appear [24]. Variables considered in this analysis were: geographical location of cases, age (grouped in three categories, children: 1 to 5 years, infant: 6 to 15, and adult: 16 or more), gender (male or female), parasite load (without parasites, low, medium, high), gametocyte presence, date (year), season (rainy or dry) and presumed origin of infection (autochthonous, including the malaria case origin province/comarca, or imported, based solely on the patients travel history).

### 2.5. Ethical Statement

Since the resurgence of malaria in Panamá between 2002 and 2004, the DCV-MoH and ICGES began a coordinated molecular surveillance of malaria cases with the goal of detecting and characterizing parasites that carry genetic markers associated with drug resistance [11]. Retrospective molecular analysis of the samples and the protocols used for molecular surveillance were approved by the DCV-MoH (No. 374/DCV/ICG) and by the Comité de Bioética de la Investigación del Instituto Conmemorativo Gorgas de Estudios de la Salud (No. 468/CNBI/ICGES/06 and No. 413/CNBI/ICGES/12). The search of suspected cases, and the diagnostic, treatment and documentation procedures of each malaria case (detected via active and passive search) were conducted by the DCV-MoH as part of the routine surveillance system for malaria control. Epidemiological information was also obtained from the DCV-MoH databases. The confidentiality of the study subjects with malaria was protected.

## 3. Results

The studied population included 106 *P. falciparum*-confirmed blood samples collected in Panama between 2003 and 2019 as part of routine malaria surveillance. From this total number of samples, 81 (76.4%) were successfully amplified for *glurp*, 82 (77.4%) for *msp*-1 and 101 (95.22%) for *msp*-2. It was possible to genotype 78 (73.6%) samples with the three combined genes. Among these 78 samples, 23 (~29.9%) were preliminarily considered as imported cases based solely on their travel history.

### 3.1. Allelic Frequency of glurp, msp-*1* and msp-*2*

**Figure 2:**
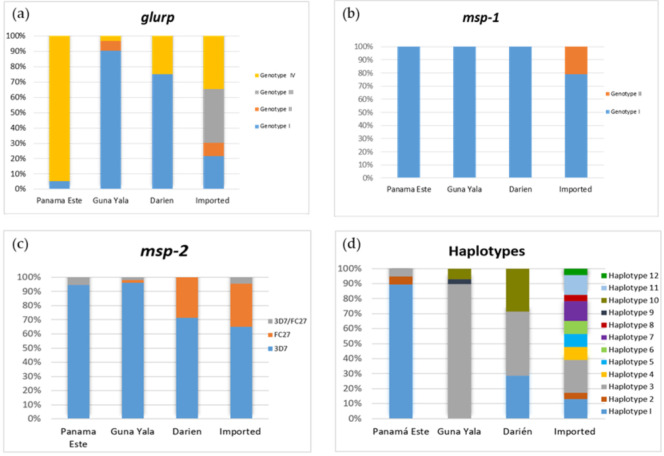
Allelic frequencies of *glurp* (**a**), *msp*-1 (**b**) and *msp*-2 (**c**) genes and haplotypes (**d**) observed in *Plasmodium falciparum* malaria endemic regions from Panamá and in imported cases based on patients’ travel history.

Four different alleles were identified for the *glurp* RII repeat region, coded as Genotypes I–IV. Fragment size ranged from 800 to 1100 bp. Genotypes I (800 bp) and IV (1100 bp) were the most common, present in 49.4% and 35.8%, respectively, of the samples analyzed for this marker. The other two genotypes (II and III) were identified in 12 (14.8%) samples, mostly related to imported cases (Figure 2 and Figure S1 and Table S1). No polyclonal infections were observed for this marker. Only two alleles were detected for the *msp*-1 marker based on the amplified fragment sizes. Most samples (93.9%; 77/82) harbored *msp*-1 Genotype I (500 bp), and only five samples from suspected imported cases displayed Genotype II (600 bp) (Figure 2 and Figure S1 and Table S1).

Restriction profile analysis of the *msp*-2 gene revealed that both allelic families were present in the studied samples. A high proportion of samples carried the 3D7 allelic family (87.1%). Ten samples (9.9%) harbored the FC27 family, from which seven were of imported origin. Three samples carried both allelic families (Figure 2 and Table S1). In the autochthonous infections, no allelic variants were detected within each family type based on the size of the variable fragments observed in the restriction profile. However, in the assumed imported cases, polymorphisms were observed with banding patterns difficult to interpret with our visual detection method. Thus, for this *msp*-2-RFLP approach, genotypes were assigned to the allelic family level. Only single infections were observed for *glurp* and *msp*-1 alleles, and three patients were detected by PCR-RFLP to have multiclonal infections at the *msp*-2 genes (3.0%).

### 3.2. Haplotype Diversity and Distribution

Combining the three genes, from 78 samples with data available on the three loci, 12 different haplotypes were identified (here named Haplotypes 1–12). Haplotype 1 (28.2%; 22/78) and Haplotype 3 (44.84%; 35/78) were the most prevalent and widespread. Haplotype 3 was detected in indigenous patients from the three *P. falciparum* endemic regions in Panamá, as well as in imported cases from different continents (Table 1, Tables S2 and S3, Figure 2). Two haplotypes were found exclusively among indigenous malaria cases from the Guna Yala and Darién regions (H9 and H10). Interestingly, the haplotype H2 was only detected in one imported case from Central Africa and in one indigenous fatal case from Panamá Este (PF01) during the 2004 epidemic when a resurge of malaria was declared in the country with the establishment of chloroquine-resistant *P. falciparum* transmission in the eastern region [7]. The other haplotypes (H4, H5, H6, H7, H8, H11 and H12) were distributed solely among presumed imported cases based on the travel histories declared by the patients (Table 1, Tables S2 and S3, Figure 2).

### 3.3. Multiple Correspondence Analysis

**Figure 3:**
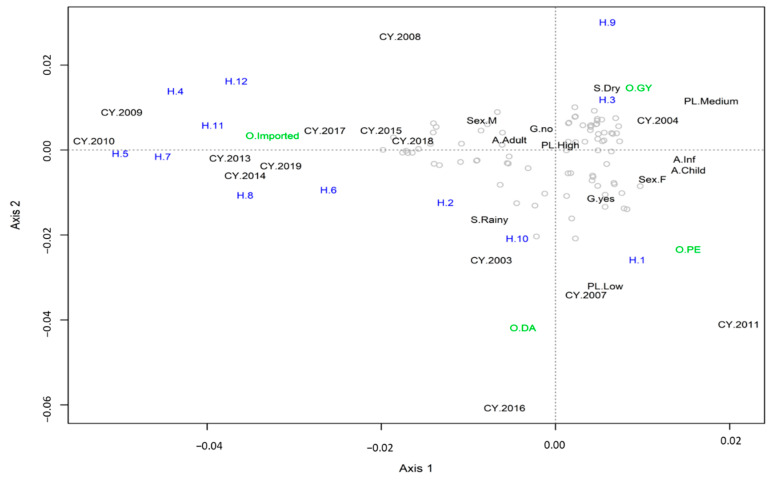
Multiple correspondence analysis. In the plot, the coordinates for each studied subject are presented by gray circles, and the dashed lines cross at the origin of the plot. Text in black indicates the centroids for the different categories of the studies variables which included: A (Age) (Child, Inf: Infant, Adult); Sex (M: Male, F: Female), S (Season) (Rainy, Dry), G (Gametocyte Presence) (Yes or No), PL (Parasite Load) (High, Medium, Low and NP for no parasites); O (Origin) (D: Darien, PE: Panamá Este, GY: Guna Yala, Imported); CY (Collection Year); and Haplotype (1 to 12). The correlation for Axis 1 was 0.611 and for Axis 2 it was 0.579, with both axes explaining up to 17.10% of the variance in the data. To ease visualization, Axis 2 coordinates of CY 2010, 2018 and 2019 were changed by, respectively, adding the following values: +0.002, −0.0015 and +0.002.

The MCA analysis showed that high parasitic loads in samples from male adults and without gametocytes were evenly distributed across the studied samples (Figure 3). Haplotypes 4, 5, 6, 7, 8, 11 and 12 were exclusively imported. These haplotypes were mainly observed in 2009 (H4), 2010 (H5) and across several years (2008, 2009, 2010, 2013, 2014, 2015, 2017, 2018 and 2019) for H6, H7, H8, H11 and H12. Haplotype H10 was primarily associated with the rainy season of 2003 and detected in Darien. Haplotype H1 was strongly associated with samples from Panamá Este taken in 2007 and 2011 in patients with low parasitemia and with gametocytes. Haplotypes H3 and H9 were strongly associated with the Guna Yala region, collected in female children and infants, with medium parasite loads in the dry season of 2004.

### 3.4. Msp-*2* Sequencing and Phylogenetic Analysis

Allelic frequencies for the *msp*-2 gene were further analyzed by sequencing the nested PCR products [20]. DNA sequencing of the *msp*-2 block 3 region was successful in 42 samples from this study: 33 considered indigenous and 9 imported based on epidemiological data from the DCV-MoH. The size of the sequenced fragments ranged between 400 and 600 bp. Initial phylogenetic analysis clearly separated both *msp*-2 allelic families: 9 samples for FC27 and 33 samples for 3D7. Phylogenetic tress for FC27 and 3D7 were constructed and analyzed separately.

**Figure 4:**
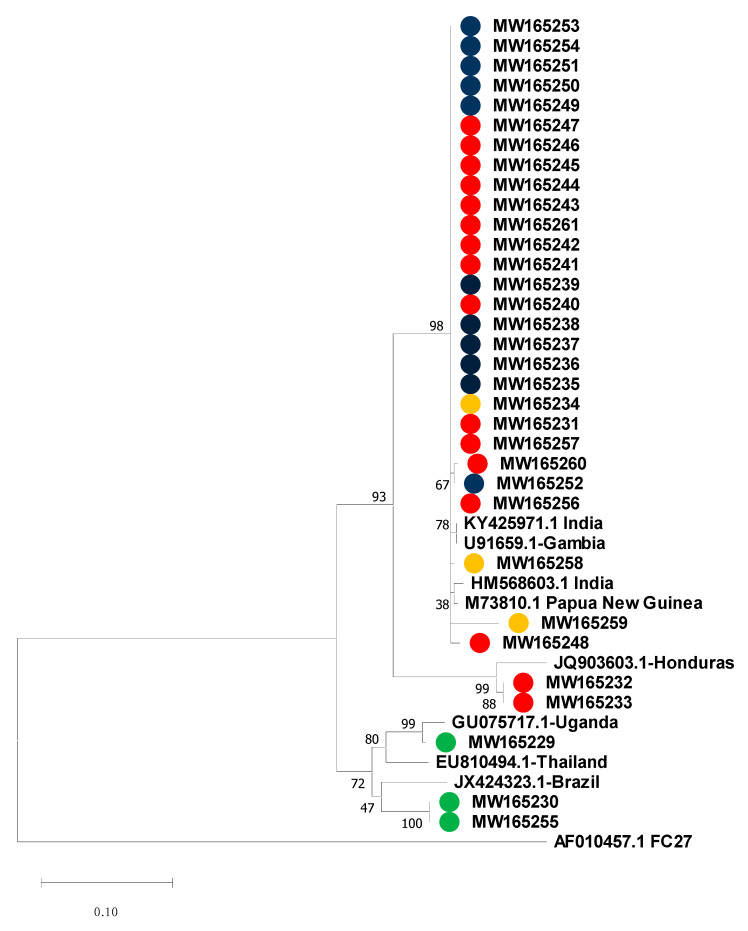
Phylogenetic tree constructed from *Plasmodium falciparum msp*-2 gene block 3, 3D7 allelic family nucleotide sequences. Sequences from this study are in colored circles (green: imported; red: Panama Este; yellow: Darien, and blue: Guna Yala). The evolutionary history was inferred by using the maximum likelihood method and general time reversible model. The tree with the highest log likelihood (−2022.53) is shown with a discrete gamma distribution used to model evolutionary rate differences among sites (5 categories (+G, parameter = 1.0259)). The tree is drawn to scale, with branch lengths measured in the number of substitutions per site. This analysis involved 42 nucleotide sequences. There were 450 positions in the final dataset. Bootstrap values were calculated for 1000 replications. Reference sequences are identified with their accession numbers and geographical location of parasite isolates. The tree was rooted using the reference sequence (AF0110457.1) of msp-2 FC27 as an out-group.

**Figure 5:**
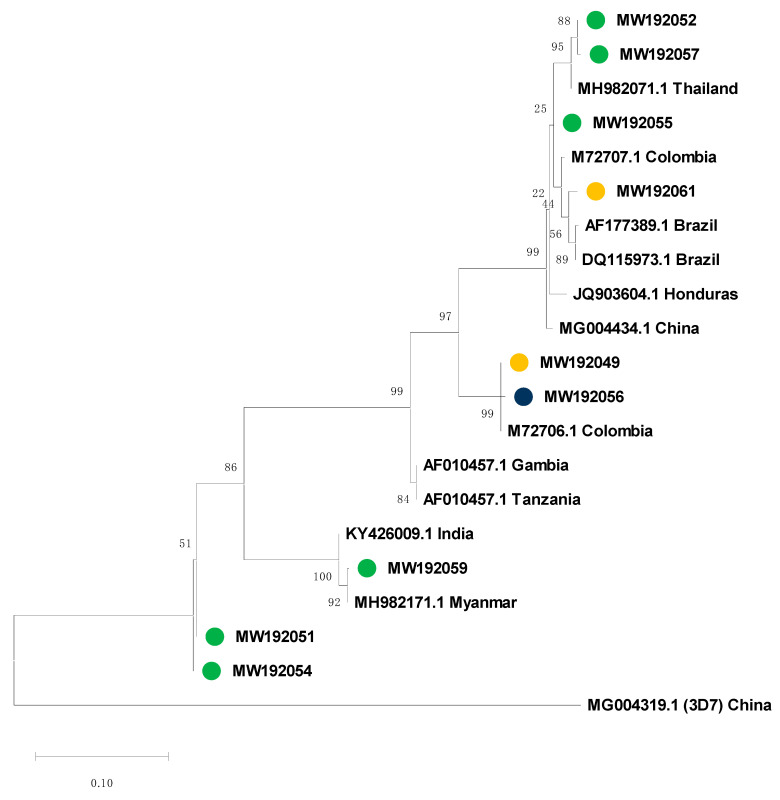
Phylogenetic tree constructed from *Plasmodium falciparum msp*-2 gene block 3, FC27 allelic family nucleotide sequences. Sequences from this study (MW192049, MW192051, MW192052, MW192054, MW192055, MW192056, MW192057, MW192059, MW192061) are in colored circles (green: imported; yellow: Darien, and blue: Guna Yala). The evolutionary history was inferred by using the maximum likelihood method based on the Tamura–Nei model. The tree with the maximum log likelihood (−885.3150) is shown. The percentage of trees in which the associated taxa clustered together is shown next to the branches. The tree is drawn to scale, with branch lengths measured in the number of substitutions per site. Bootstrap values were calculated for 1000 replications. This analysis involved 21 nucleotide sequences. Reference sequences are identified with their accession numbers and geographical location of parasite isolates. The tree was rooted using the reference sequence (MG004319.1) of msp-2 3D7 as an out-group.

To determine the genetic relatedness between the *P. falciparum* samples evaluated in this study, the msp-2 partial sequences were aligned and compared with previously published *msp*-2 sequences from Latin America and from various geographic localities where the presumed imported cases originated. The final phylogenetic analysis contained 63 sequences, including 42 from this study and 21 reference sequences accessed from GenBank (Figure 4 and Figure 5). Complete nucleotides alignment for both allelic families is available upon request.

The phylogenetic tree from sequences that belong to 3D7 showed that representative indigenous isolates from the three *P. falciparum* endemic regions in Panamá (GenBank accession number MW165229-MW165261) grouped together with other reference sequences from Asia, Africa and South America (Figure 4). Only two indigenous *P. falciparum* isolates from Panamá Este (MW165232 and MW165233) were closely related to the only reference sequences representative for the Mesoamerican region (Honduras, JQ903603.1), suggesting that some *P. falciparum* populations circulating in Panamá might have different genetic structures compared to the rest of the region. Within the FC27 analysis, local cases (MW192049, MW192056 and MW192061) grouped together with South American reference samples from Colombia and Brazil and apart from branches containing samples from India and Myanmar (Figure 5). No geographic clustering was observed among indigenous samples. The presumed imported cases were distributed in different branches related to the geographical origin. In general, our phylogenetic analysis confirmed the travel history of most imported cases.

## 4. Discussion

In line with regional agreements, Panamá has committed to eliminate malaria from all of its territory by 2020 [1,25]. As the country moves towards this regional goal, many political, cultural and biological challenges already identified still need to be properly addressed [1,7,26,27]. In this regard, high-transmission areas where malaria has historically persisted have already been identified and can now be targeted with appropriate interventions to reduce transmission [1,7,15]. Equally important for the elimination goal is the assessment of imported malaria risk [28]. Panamá, as a biological corridor joining South America with the rest of the Mesoamerican region, has permeable boundaries that render the nation vulnerable to the entrance of migrants, some of them intending to reach the United States or Canada. During the last decade, the demographic profile of Panama–Colombia cross-borders has considerably diversified, with growing numbers originating from Africa and Asia [7,29]. Both regions are known for the circulation of multidrug-resistant *Plasmodium* spp. strains, posing an additional challenge, not only to Panamá’s national malaria program, but also to the regional Mesoamerican malaria elimination plan [25]. Imported malaria cases by *P. falciparum* and *P. vivax* have been confirmed in travelers entering Panamá from South America. During the last decade, more *P. falciparum* imported than autochthonous cases have been detected in the country [7]. Moreover, *P. falciparum* reintroduction in eastern areas of the country has been epidemiologically related to an imported origin [10,13]. Previous studies have described that refugees and migrants from Africa to North America are frequently infected with malaria, predominantly asymptomatic *P. falciparum* infections [29,30,31], and asymptomatic patients can act as reservoirs and provoke malaria epidemics in areas where the disease had previously been eliminated [32].

In general, the data and design of this study do not support the claim that *P. falciparum* local cases originated from imported infections. However, an active molecular surveillance to identify genotypes circulating along the border areas and an efficient cooperation between countries of the region are particularly needed for monitoring malaria control interventions and understanding parasite dynamics related to imported cases [28,33]. This is particularly true now that Mesoamerican countries are actively involved in a malaria elimination campaign, and that the introduction of *P. falciparum* chloroquine-resistant strains coupled with a high transmission vulnerability [34] of this region would be a major drawback for this regional purpose.

In this context, the present study has identified a relatively high diversity in Panamanian samples using three conventional molecular genes recommended to genotype *P. falciparum* (*glurp*, *msp*-1 and *msp*-2). However, when these samples were disaggregated based on the travel history of the patients, imported samples accounted for most of the observed genetic diversity. In fact, when combining the three genes, most indigenous samples grouped in only two of the 12 haplotypes identified in this study (H1 and H3)—a finding consonant with the low transmission of malaria in the country [7] and the clonal structure of local infections described in previous studies [10]. Importantly, some of these haplotypes were exclusively found in indigenous cases (H9, H10), while others (H4, H5, H6, H7, H8, H11 and H12) were only observed among imported cases (Table 1, Tables S2 and S3, Figure 3). However, due to the low number of indigenous samples analyzed in this study, we cannot rule out the presence of these haplotypes in local cases. Within indigenous samples, the MCA analysis not only evidenced a strong association of certain local haplotypes with their geographic distribution in endemic areas, but also with relevant epidemiological parameters such as parasite load and presence of gametocytes (Figure 3 and Table S3). Altogether, this genetic information is key to understanding the dynamics of disease transmission in the country and to develop effective methods to trace the origin of infections by the NMEP towards the elimination goal.

In contrast with the rest of Mesoamerica, during recent decades, *P. falciparum* parasites circulating in Panamá have been subjected to different drug pressures mostly because of changes in treatment policies based on clinical and molecular findings [9,11,12]. This situation might have shaped the genetic structure of *P. falciparum* in the country, and might explain why, except for Panama, Mesoamerica is remarkably the only region in the world were autochthonous *P. falciparum* parasites are still sensitive to CQ. Not surprisingly, the phylogenetic analysis did not group the Panamanian *P. falciparum* samples with other Mesoamerican reference isolates (Figure 4). This could also reflect the effects of immune-mediated balancing selection, which tends to decrease divergence between populations relative to genes under directional or purifying selection [35]. However, this pattern might be due to a lack of sequence data from Mesoamerican samples in our phylogenetic analysis. The limited number of *msp*-2 *P. falciparum* reference sequences from Mesoamerica, available in public genetic databases, might be due to the low transmission of *P. falciparum* when compared to *P. vivax* in Mesoamerica, but it also might reflect the scarcity of groups conducting molecular epidemiology research in Mesoamerica. In one of these studies, a similar low diversity was identified for the *msp*-2 gene in eleven *P. falciparum* isolates from Honduras in Central America, all of them from the 3D7 family [36].

Knowing the geographical area where a patient becomes infected with *Plasmodium* spp. and genotyping the parasites causing infections are essential to design and target elimination policies. While the WHO recommends malaria infection origin classification as imported based on times since potential parasite exposure, it is sometimes difficult to distinguish between local and imported malaria based solely on the travel history. In many cases, because of the fear of deportation and/or language barriers, extra-continental migrants are reluctant to describe the route they used to enter the Panamanian border. Frequently, they enter South America via Ecuador and Brazil, countries with relaxed visa requirements. From there, they begin a long and difficult journey through several malaria endemic landscapes before reaching North America as their final destination [29]. In these situations, phylogenetic analysis can provide additional information regarding the geographical origin and relatedness between *Plasmodium spp*. infections in this mobile population. In this regard, our phylogenetic analysis based on the *msp*-2 gene, in most cases, corroborates the imported status of the *P. falciparum* infections that were presumed initially on their travel history (Figure 4 and Figure 5).

Of the three molecular markers analyzed, *glurp* showed individually the highest diversity in both autochthonous and imported samples, while *msp*-1 was the least polymorphic gene. The better performance of *glurp* to detect genetic diversity in different epidemiological settings has been previously described [37]. In fact, in this study, *msp*-1 and *msp*-2 showed low resolution power due to an almost geographically uniform genotype in non-imported cases (Tables S1 and S3). In this line, it is important to mention that a drawback of this study, and previous studies using similar molecular protocols [19,20,21,22,37], is the assignment of allelic variants based on amplified DNA fragments size based on observations from electrophoretic migration in gels. Factors such as gel quality, resolution of the method used to analyze amplified fragments and visual vs. digital analysis can all add some degree of subjectivity to the calling of alleles [37]. In our study, this was particularly troublesome when analyzing the variable fragments in the *msp-2* RFLP analysis. This is one of the reasons why we further sequenced this gene for a more reliable approach to assign an allele within each allelic family. A further limitation of this study was the lack of a sample size for a reliable determination of *P. falciparum* diversity in Panamá.

As countries of Mesoamerica approach malaria elimination and local transmission decreases, more emphasis will be needed in tackling the threat of imported malaria. At present, four countries in Mesoamerica (Costa Rica, El Salvador, Belize and Mexico) have been identified by the WHO as having the potential to soon eliminate malaria [38]. In these countries, as in Panamá, an important proportion of cases are found among migrant and mobile populations living in remote areas mostly near the borders [39,40,41,42]. For regional elimination efforts to succeed, imported infections must be detected and treated rapidly. This involves an effective and coordinated regional collaboration, including the implementation of standard, practical and affordable genotyping protocols for conducting a reliable comparative analysis across countries, in order to accurately infer infection origin, drug resistance and disease propagation patterns in the region.

## 5. Conclusions

This study has shown few multiclonal infections and low genetic diversity among locally transmitted *P. falciparum* samples, echoing the low transmission intensity of this parasite observed in Panamá, a pattern likely to be extended across Mesoamerica. In addition, several imported cases, as expected, were genetically dissimilar to local infections and representative of more diverse extra-continental lineages.

## Figures and Tables

**Table 1:** Haplotypes diversity and distribution inferred by combining the alleles detected in the three loci (*glurp*, *msp*-1 and *msp*-2) in *Plasmodium falciparum* indigenous and imported cases based on travel history.

Haplotype	Gene Alleles			Case Origin			No	Total
	*glurp*	*msp*-2	*msp*-1	Indigenous	No	Imported		
1	1100 bp	3D7	500 bp	Panamá Este	17	Africa	3	22
				Darién	2			
2	1100 bp	3D7/FC27	500 bp	Panamá Este	1	Central Africa	1	2
3	800 bp	3D7	500 bp	Guna Yala	26	South America *	1	
				Darién	3	Egypt	1	
				Panamá Este	1	Tanzania	1	35
						Zimbabwe	1	
						Africa **	1	
								
4	1100 bp	FC27	500 bp			Haiti	1	2
						Zimbabwe	1	
5	1000 bp	FC27	500 bp			Philippines	1	2
						India	1	
								
6	900 bp	FC27	500 bp			China	1	2
						Africa	1	
								
7	1000 bp	3D7	500 bp			Colombia	1	3
						Nigeria	1	
						Equatorial Guinea	1	
								
8	1100 bp	3D7	500 bp			West Africa	1	1
								
9	800 bp	3D7/FC27	500 bp	Guna Yala	1			1
								
10	800 bp	FC27	500 bp	Darién	2			4
				Guna Yala	2			
11	1000 bp	3D7	600 bp			Cameroon	2	3
						India	1	
12	1100 bp	FC27	600 bp			Cameroon	1	1
Total					55		23	78

* Patient reported to have traveled recently to many countries in South America (Venezuela/Guyana /Colombia) before entering Panamá via Guna Yala. ** Patient reported to have traveled recently to the Philippines and Mozambique.

## Supplementary Materials

Supplementary materials can be found at https://www.mdpi.com/2075-1729/10/12/319/s1. Figure S1: Genotyping of *Plasmodium falciparum* infections by PCR amplification of: (a) Merozoite surface proteins 1 (*msp*-1), (b) Merozoite surface proteins 2 (*msp*-2) and (c) Glutamate rich protein (*glurp*), Table S1: Prevalence of genotypes (*msp*-1, *msp*-2 and *glurp* alleles) by origin of the infection in patients from Panamá. Alleles of *glurp* and *msp*-1 were classified by fragment length of the nested PCR products. Allelic families of *msp*-2 were identified by PCR-RFLP (Hinf I), Table S2: Haplotypes diversity and distribution inferred by combining the alleles detected in the three loci (*glurp*, *msp*-2 and *msp*-1) in *Plasmodium falciparum* indigenous and imported cases based on patients´ travel history, Table S3: Epidemiological data and genotyping results from samples analyzed in this study.

## Abbreviations

ACT	Artemisinin-based combination therapy
CQ	Chloroquine
DCV-MoH	Departamento de Control de Vectores from the Ministry of Health
*glurp*	Glutamate-rich protein
ICGES	Instituto Conmemorativo Gorgas de Estudios de la Salud
MCA	Multiple correspondence analysis
*msp*-1	Merozoite surface proteins 1
*msp*-2	Merozoite surface proteins 2
MOI	Multiplicity of infection
NMEP	National Malaria Elimination Programme
RFLP	Restriction fragment length polymorphism
SP	Sulfadoxine–pyrimethamine

## References

[1] Ministerio de Salud (2018). Plan Estratégico de Eliminación de la Malaria (PEEM) en Panamá 2018–2022.

[2] Obaldia N. (2015). Determinants of low socio-economic status and risk of *Plasmodium vivax* malaria infection in Panama (2009–2012): A case-control study. Malar. J..

[3] Hurtado L.A., Calzada J.E., Rigg C.A., Castillo M., Chaves L.F. (2018). Climatic fluctuations and malaria transmission dynamics, prior to elimination, in Guna Yala, Republica de Panamá. Malar. J..

[4] Rigg C.A., Hurtado L.A., Calzada J.E., Chaves L.F. (2019). Malaria infection rates in *Anopheles albimanus* (Diptera: Culicidae) at Ipetí-Guna, a village within a region targeted for malaria elimination in Panamá. Infect. Genet. Evol..

[5] Cáceres-Carrera L., Victoria C., Ramirez J.L., Jackman C., Calzada J.E., Torres R. (2019). Study of the epidemiological behavior of malaria in the Darien Region, Panama. 2015–2017. PLoS ONE.

[6] Hurtado L.A., Rigg C.A., Calzada J.E., Dutary S., Bernal D., Koo S.I., Chaves L.F. (2018). Population Dynamics of *Anopheles albimanus* (Diptera: Culicidae) at Ipetí-Guna, a Village in a Region Targeted for Malaria Elimination in Panamá. Insects.

[7] Hurtado L., Cumbrera A., Rigg C., Perea M., Santamaría A.M., Chaves L.F., Moreno D., Romero L., Lasso J., Caceres L. (2020). Long-term transmission patterns and public health policies leading to malaria elimination in Panamá. Malar. J..

[8] Samudio F., Santamaría A.M., Obaldía N., Pascale J.M., Bayard V., Calzada J.E. (2005). Prevalence of *Plasmodium falciparum* mutations associated with antimalarial drug resistance during an epidemic in Kuna Yala, Panama, Central America. Am. J. Trop. Med. Hyg..

[9] Calzada J.E., Samudio F., Bayard V., Obaldia N., de Mosca I.B., Pascale J.M. (2008). Revising antimalarial drug policy in Central America: Experience in Panama. Trans. R. Soc. Trop. Med. Hyg..

[10] Obaldia N., Baro N.K., Calzada J.E., Santamaria A.M., Daniels R., Wong W., Chang H.H., Hamilton E.J., Arevalo-Herrera M., Herrera S. (2015). Clonal outbreak of *Plasmodium falciparum* infection in eastern Panama. J. Infect. Dis..

[11] Ministerio de Salud Panamá (2011). Manual de Normas y Procedimientos para Malaria.

[12] World Health Organization (2015). Guidelines for the Treatment of Malaria.

[13] Calzada J.E., Marquez R., Rigg C., Victoria C., De La Cruz M., Chaves L.F., Cáceres L. (2015). Characterization of a recent malaria outbreak in the autonomous indigenous region of Guna Yala, Panama. Malar. J..

[14] Herrera S., Ochoa-Orozco S.A., González I.J., Peinado L., Quiñones M.L., Arévalo-Herrera M. (2015). Prospects for malaria elimination in Mesoamerica and Hispaniola. PLoS Negl. Trop. Dis..

[15] Lainhart W., Dutari L.C., Rovira J.R., Sucupira I.M., Póvoa M.M., Conn J.E., Loaiza J.R. (2016). Epidemic and Non-Epidemic Hot Spots of Malaria Transmission Occur in Indigenous Comarcas of Panama. PLoS Negl. Trop. Dis..

[16] Kotepui M., Piwkham D., PhunPhuech B., Phiwklam N., Chupeerach C., Duangmano S. (2015). Effects of malaria parasite density on blood cell parameters. PLoS ONE.

[17] Snounou G., Viriyakosol S., Jarra W., Thaithong S., Brown K.N. (1993). Identification of the four human malaria parasite species in field samples by the polymerase chain reaction and detection of a high prevalence of mixed infections. Mol. Biochem. Parasitol..

[18] Snewin V.A., Herrera M., Sanchez G., Scherf A., Langsley G., Herrera S. (1991). Polymorphism of the alleles of the merozoite surface antigens MSA1 and MSA2 in *Plasmodium falciparum* wild isolates from Colombia. Mol. Biochem. Parasitol..

[19] Ranford-Cartwright L.C., Taylor J., Umasunthar T., Taylor L.H., Babiker H.A., Lell B., Schmidt-Ott J.R., Lehman L.G., Walliker D., Kremsner P.G. (1997). Molecular analysis of recrudescent parasites in a *Plasmodium falciparum* drug efficacy trial in Gabon. Trans. R. Soc. Trop. Med. Hyg..

[20] Felger I., Irion A., Steiger S., Beck H.P. (1999). Genotypes of merozoite surface protein 2 of *Plasmodium falciparum* in Tanzania. Trans. R. Soc. Trop. Med. Hyg..

[21] Kidima W., Nkwengulila G. (2015). *Plasmodium falciparum* msp2 Genotypes and Multiplicity of Infections among Children under Five Years with Uncomplicated Malaria in Kibaha, Tanzania. J. Parasitol. Res..

[22] Felger I., Beck H.P., Doolan D.L. (2002). Genotyping of *Plasmodium falciparum*. Malaria Methods and Protocols: Methods in Molecular Medicine.

[23] Venables W.N., Ripley B.D. (2002). Modern Applied Statistics with S.

[24] Rigg C.A., Calzada J.E., Saldaña A., Perea M., Chaves L.F., Valderrama A. (2019). *Leishmania* spp. Infection Rate and Feeding Patterns of Sand Flies (Diptera: Psychodidae) from a Hyperendemic Cutaneous Leishmaniasis Community in Panamá. Am. J. Trop. Med. Hyg..

[25] Consejo de Ministros de Salud de Centroamérica y República Dominicana (COMISCA) (2013). Declaración-Hacia la Eliminación de la Malaria en Mesoamérica y la Isla de la Española en el 2020.

[26] Hurtado L.A., Cáceres L., Chaves L.F., Calzada J.E. (2014). When climate change couples social neglect: Malaria dynamics in Panamá. Emerg. Microbes Infect..

[27] Cáceres L., Calzada J.E., Gabster A., Young J., Márquez R., Torres R., Griffith M. (2017). Social representations of malaria in the Guna indigenous population of Comarca Guna de Madungandi, Panama. Malar. J..

[28] Sturrock H.J.W., Roberts K.W., Wegbreit J., Ohrt C., Gosling R.D. (2015). Tackling imported malaria: An elimination endgame. Am. J. Trop. Med. Hyg..

[29] Yates C. As More Migrants from Africa and Asia Arrive in Latin America, Governments Seek Orderly and Controlled Pathways. https://www.migrationpolicy.org/article/extracontinental-migrants-latin-america.

[30] Ndao M., Bandyayera E., Kokoskin E., Gyorkos T.W., MacLean J.D., Ward B.J. (2004). Comparison of blood smear, antigen detection, and nested-PCR methods for screening refugees from regions where malaria is endemic after a malaria outbreak in Quebec, Canada. J. Clin. Microbiol..

[31] Monge-Maillo B., López-Vélez R. (2011). Is screening for malaria necessary among asymptomatic refugees and immigrants coming from endemic countries?. Expert Rev. Anti-Infect. Ther..

[32] Martens P., Hall L. (2000). Malaria on the move: Human population movement and malaria transmission. Emerg. Infect. Dis..

[33] World Health Organization (2017). A Framework for Malaria Elimination.

[34] Hotez P.J., Woc-Colburn L., Bottazzi M.E. (2014). Neglected tropical diseases in Central America and Panama: Review of their prevalence, populations at risk and impact on regional development. Int. J. Parasitol..

[35] Van Tyne D., Park D.J., Schaffner S.F., Neafsey D.E., Angelino E., Cortese J.F., Barnes K.G., Rosen D.M., Lukens A.K., Daniels R.F. (2011). Identification and functional validation of the novel antimalarial resistance locus PF10_0355 in *Plasmodium falciparum*. PLoS Genet..

[36] Lopez A.C., Ortiz A., Coello J., Sosa-Ochoa W., Torres R.E.M., Banegas E.I., Jovel I., Fontecha G.A. (2012). Genetic diversity of *Plasmodium vivax* and *Plasmodium falciparum* in Honduras. Malar. J..

[37] Medicines for Malaria Venture & World Health Organization (2007). Methods and Techniques for Clinical Trials on Antimalarial Drug Efficacy: Genotyping to Identify Parasite Populations.

[38] World Health Organization (2018). Update on the e-2020 Initiative of 21 Malaria-Eliminating Countries: Report and Country Briefs.

[39] Bennett A., Smith J.L. (2018). Malaria Elimination: Lessons from El Salvador. Am. J. Trop. Med. Hyg..

[40] Marín Rodríguez R., Chaves L.F. (2019). Parasite Removal for Malaria Elimination in Costa Rica. Trends Parasitol..

[41] Chaves L.F., Huber J.H., Rojas Salas O., Ramírez Rojas M., Romero L.M., Gutiérrez Alvarado J.M., Perkins T.A., Prado M., Marín Rodríguez R. (2020). Malaria Elimination in Costa Rica: Changes in Treatment and Mass Drug Administration. Microorganisms.

[42] Chaves L.F., Ramírez Rojas M., Prado M., Garcés J.L., Salas Peraza D., Marín Rodríguez R. (2020). Health policy impacts on malaria transmission in Costa Rica. Parasitology.

