# Supplementary material for: Plasmodium falciparum Genetic Diversity in Panamá Based on glurp, msp-1 and msp-2 Genes: Implications for Malaria Elimination in Mesoamerica
Source: Life (Basel). 2020 Nov 28;10(12):319. doi: 10.3390/life10120319 (PMC7760695; doi:10.3390/life10120319)
Supplement: Supplementary file 1 [file life-10-00319-s001.pdf]

Supplementary Materials

Plasmodium falciparum Genetic Diversity in Panamá Based on *glurp, msp*-1 and *msp*-2 genes: Implications for Malaria Elimination in Mesoamerica

(A)

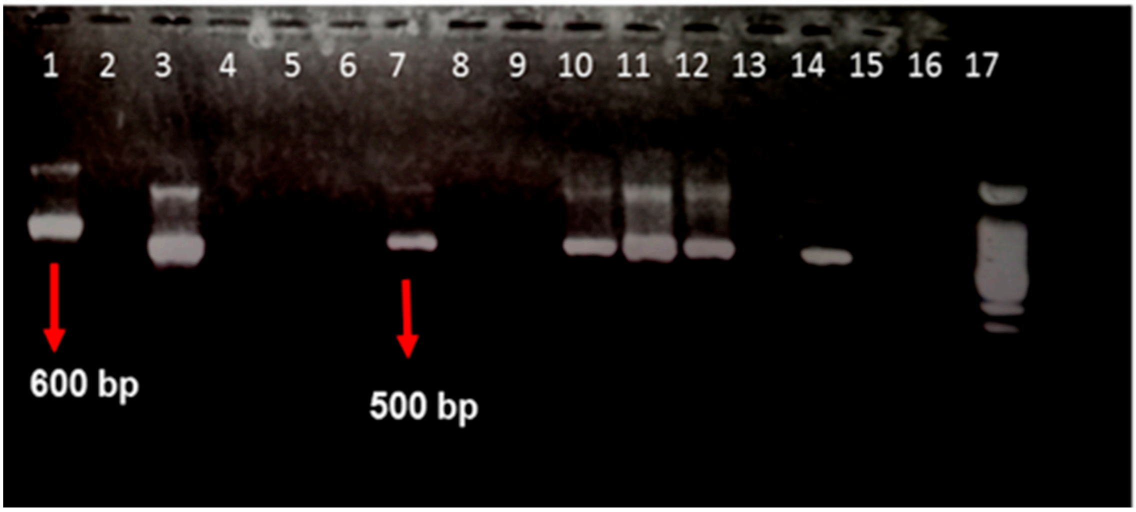

(B)

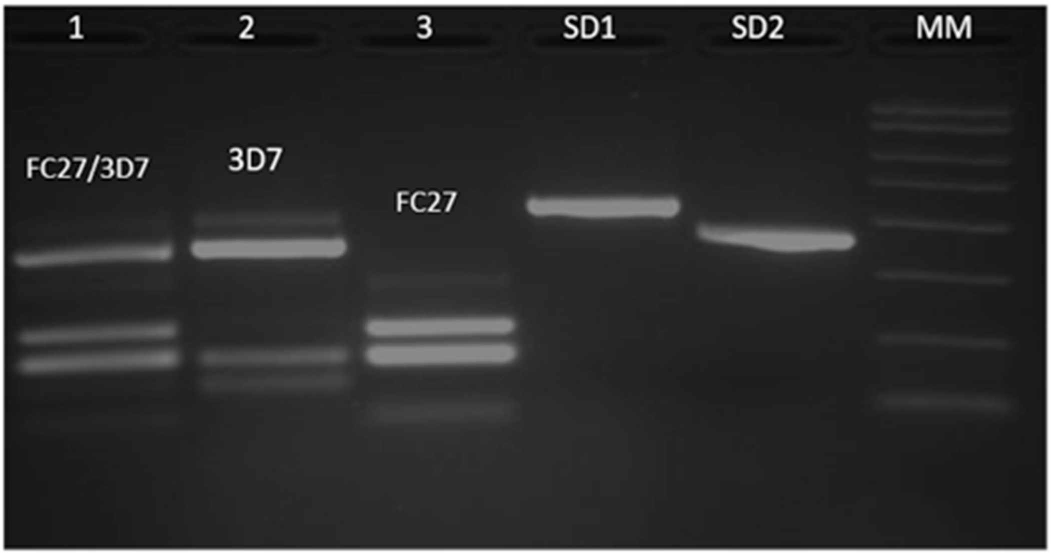

(C)

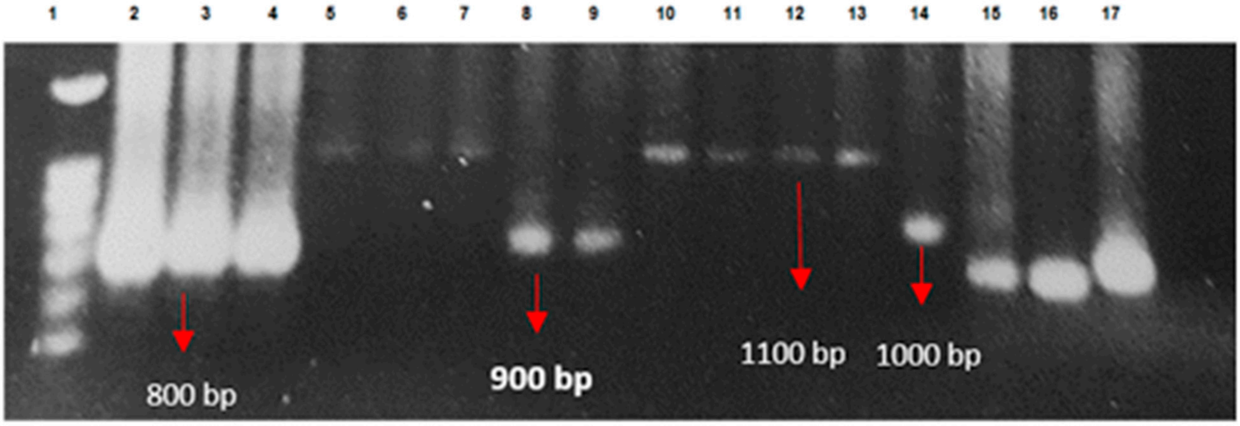

Figure S1. Genotyping of *Plasmodium falciparum* infections by PCR amplification of: (**a**). Merozoite surface proteins 1 (msp-1), (**b**) Merozoite surface proteins 2 (msp-2) and (**c**) Glutamate rich protein (glurp).

(a) Representative agarose gel for *P. falciparum msp*-1 gene nested PCR products. Lane 1: 600 bp genotype. Lanes 3, 7, 10, 11, 12 and 14: 500 bp genotype; Lanes 2, 4, 5, 6 8, 9, 13,15 and 16: no amplification; Lane 17: Molecular weight marker (100-bp ladder).

(b) Representative agarose gel for *P. falciparum msp*-2 gene products digested with Hinf I. Lane 1: 3D7/FC27 mixed infection; Lane 2: Allelic family 3D7; Lane 3: Allelic Family FC27; SD1 and SD2: Undigested PCR product; MM: Molecular weight marker (100-bp ladder).

(c) Representative agarose gel for *P. falciparum glurp* gene nested PCR products. Lane 1: Molecular weight marker (100-bp ladder); Lanes 2, 3, 4, 15, 16, and 17: 800 bp genotype; Lanes 5, 6, 7,10,11,12 and 13: 1100 bp genotype; Lanes 8 and 9: 900 bp genotype; Lane 14: 1000 bp genotype.

Table S1. Prevalence of genotypes (*msp-1*, *msp-2* and *glurp* alleles) by origin of the infection in patients from Panamá. Alleles of *glurp* and *msp-1* were classified by fragment length of the nested PCR products. Allelic families of msp-2 were identified by PCR-RFLP (Hinf I).

Isolates Origin	*glurp* Genotype I	*glurp* Genotype II	*glurp* Genotype III	*glurp* Genotype IV	*msp-1* Genotype I	*msp-1* Genotype II	*msp-2* (3D7)	*msp-2* (FC27)	*msp-2* (3D7/FC27)
**Panamá Este**	1 (1.2%)	0	0	18 (22.2%)	19 (23.2%)	0	18 (17.8%)	0	1 (1.0%)
**Guna Yala**	28 (34.6%)	2 (2.5%)	0	1 (1.2%)	31 (37.8%)	0	50 (49.5%)	1 (1.0%)	1 (1.0%)
**Darién**	6 (7.4%)	0	0	2 (2.5%)	8 (9.8%)	0	5 (5.0%)	2 (2.0%)	0
**Imported***	5 (6.2%)	2 (2.5%)	8 (9.8%)	8 (9.8%)	19 (23.2%)	5 (6.1%)	15 (14.8%)	7 (6.9%)	1 (1.0%)
**TOTAL**	**40**	**4**	**8**	**29**	**77**	**5**	**88**	**10**	**3**
	**81**				**82**		**101**		

* Imported status was inferred by the epidemiological data provided National Malaria Programme based on the travel history declared by the patients.

Table S2. Haplotypes diversity and distribution inferred by combining the alleles detected in the three loci (*glurp*, *msp-2* and *msp-1*) in *Plasmodium falciparum* indigenous and imported cases based on patients´ travel history.

	glurp	***msp-2***	***msp-1***	**Darién**	**Guna Yala**	**Panamá Este**	**Imported**	**Total**
Haplotype 1	1100 bp	3D7	500 bp	2		17	3	22
Haplotype 2	1100 bp	3D7/FC27	500 bp			1	1	2
Haplotype 3	800 bp	3D7	500 bp	3	26	1	5	35
Haplotype 4	1100 bp	FC27	500 pb				2	2
Haplotype 5	1000 bp	FC27	500 pb				2	2
Haplotype 6	900 bp	FC27	500 pb				2	2
Haplotype 7	1000 bp	3D7	500pb				3	3
Haplotype 8	1100 bp	3D7	600 pb				1	1
Haplotype 9	800 bp	3D7/FC27	500 bp		1			1
Haplotype 10	800 bp	FC27	500 bp	2	2			4
Haplotype 11	1000 bp	3D7	600 pb				3	3
Haplotype 12	1100 bp	FC27	600 pb				1	1
				**7**	**29**	**19**	**23**	**78**

Table S3. Epidemiological data and genotyping results from samples analyzed in this study.

Number	glurp	msp-1	msp-2	H	A	Age	Sex	Locality	Province	CY	S	PL	GP	Origin
**PB01**	G4	G1	G2	1	Adult	42	F	Puente Bayano	PE	2004	Rainy	High	no	Local
**PB02**	G4	G1	G2	1	Infant	12	F	Puente Bayano	PE	2004	Rainy	Medium	yes	Local
**PB03**	G4	G1	G2	1	Adult	23	F	Puente Bayano	PE	2004	Rainy	Low	yes	Local
**PB04**	G4	G1	G2	1	Infant	7	F	Puente Bayano	PE	2004	Rainy	High	no	Local
**PB05**	G4	G1	G2	1	Infant	15	F	Puente Bayano	PE	2004	Rainy	High	no	Local
**PB06**	G4	G1	G2	1	Infant	9	F	Puente Bayano	PE	2004	Rainy	Low	yes	Local
**PB07**	G4	G1	G2	1	Adult	69	F	Puente Bayano	PE	2004	Dry	High	no	Local
**PB08**	G4	G1	G2	1	Adult	36	F	Puente Bayano	PE	2004	Rainy	High	no	Local
**PB09**	G4	G1	G2	1	Infant	7	M	Puente Bayano	PE	2004	Rainy	High	no	Local
**PB10**	G4	G1	G2	1	Child	2	F	Puente Bayano	PE	2004	Rainy	High	yes	Local
**PB11**	G4	G1	G2	1	Infant	7	F	Puente Bayano	PE	2011	Rainy	High	no	Local
**PB12**	G4	G1	G2	1	Child	3	F	Puente Bayano	PE	2004	Rainy	High	no	Local
**PB13**	G4	G1	G2	1	Child	4	M	Puente Bayano	PE	2004	Rainy	High	no	Local
**PB14**	G4	G1	G2	1	Adult	51	M	Puente Bayano	PE	2004	Rainy	High	no	Local
**PB15**	G4	G1	G2	1	Child	5	F	Puente Bayano	PE	2004	Rainy	High	no	Local
**PE01**	G4	G1	G2	1	Adult	36	F	Naragandi	PE	2007	Dry	High	yes	Local
**PM01**	G4	G1	G2	1	Adult	18	F	Río Sabalo	PE	2007	Dry	High	no	Local
**PF01**	G4	G1	G3	2	Adult	21	F	Aguas Claras, Puente Bayano	PE	2004	Rainy	High	no	Local
**PF02**	G1	G1	G1	10	Adult	34	M	Ticanti Nargana	GY	2003	Rainy	NA	no	Local
**PF03**	G1	G1	G2	3	Child	4	M	Aguas Claras, Puente Bayano	PE	2004	Dry	High	no	Local
**PF04**	G1	G1	G1	10	Adult	35	F	Jaque	DA	2003	Rainy	NA	no	Local
**DA02**	G4	G1	G2	1	Adult	41	F	Camino Real	DA	2007	Rainy	Low	yes	Local
**DA03**	G1	G1	G2	3	Infant	8	M	Puerto Piña	DA	2007	Rainy	High	yes	Local
**DA04**	G1	G1	G2	3	Adult	43	M	Puerto Piña	DA	2007	Rainy	High	yes	Local
**DA29**	G1	G1	G2	3	Adult	41	F	Jaque	DA	2007	Rainy	Low	yes	Local
**DA55**	G4	G1	G2	1	Adult	42	M	Cocalito	DA	2016	Rainy	Low	no	Local
**DA70**	G1	G1	NA	NA	Adult	21	M	Puerto Piña	DA	2019	Rainy	High	yes	Local
**DA72**	G1	G1	G1	10	Adult	37	F	Puerto Piña	DA	2019	Rainy	High	yes	Local
**PO04**	G1	G1	G2	3	Adult	19	M	Africa, Tanzania	Imported	2008	Dry	High	no	Imported
**PO08**	G4	G1	G1	4	Adult	50	M	Haiti	Imported	2009	Rainy	NA	no	Imported
**PO09**	G3	G1	G1	5	Adult	41	M	Philippines	Imported	2010	Rainy	NA	no	Imported
**PO11**	G2	G1	G1	6	Adult	18	F	China	Imported	2007	Dry	High	no	Imported

**P013**	G3	G1	G2	7	Adult	21	M	Africa (Equatorial Guinea)	Imported	2013	Rainy	High	no	Imported
**PO14**	G1	G1	G2	3	Infant	14	M	Africa (Tanzania, Kenya, South Africa)	Imported	2013	Rainy	High	no	Imported
**PO15**	G2	G1	G1	6	Adult	69	M	Africa (Mozambique) Philippines	Imported	2013	Rainy	High	yes	Imported
**PO16**	G3	G1	G1	5	Adult	25	M	India	Imported	2014	Rainy	High	no	Imported
**PO17**	G3	G1	G2	7	Adult	23	M	Africa, Nigeria	Imported	2014	Rainy	High	no	Imported
**PO18**	G4	G2	G2	8	Adult	59	F	West Africa	Imported	2014	Rainy	High	no	Imported
**PO19**	G1	G1	G2	3	Adult	34	M	Egypt	Imported	2015	Dry	High	yes	Imported
**PO20**	G3	G2	G2	11	Adult	29	M	India, Bombay	Imported	2015	Rainy	NA	no	Imported
**PO23**	G1	G1	G2	3	Adult	25	F	Guyana, Venezuela, Colombia	Imported	2015	Rainy	High	yes	Imported
**PO41**	G4	G1	G1	4	Adult	36	M	Africa (Zimbabwe, Malawi)	Imported	2017	Dry	NA	no	Imported
**PO46**	G1	G1	G2	3	Adult	36	M	Africa (Zimbabwe, Malawi)	Imported	2017	Dry	NA	no	Imported
**PO47**	NA	G1	NA	NA	Adult	36	M	Africa (Zimbabwe, Malawi)	Imported	2017	Dry	NA	no	Imported
**PO51**	G4	G1	G2	1	Adult	53	M	Africa	Imported	2017	Rainy	High	no	Imported
**PO52**	G4	G1	G3	2	Adult	54	M	Central Africa (Louma)	Imported	2017	Rainy	High	yes	Imported
**PO53**	G4	G1	G2	1	Adult	44	M	Africa (Burkina Faso)	Imported	2017	Rainy	NA	no	Imported
**PO55**	G4	G1	G2	1	Adult	41	M	Africa, Saudi Arabia	Imported	2018	Dry	NA	no	Imported
**PO57**	G3	G2	G2	11	Adult	23	M	Africa (Cameroon)	Imported	2019	Rainy	High	no	Imported
**PO58**	G3	G2	G2	11	Adult	22	M	Africa, Cameroon)	Imported	2019	Dry	High	no	Imported
**PO60**	G4	G2	G1	12	Adult	33	M	Africa (Cameroon)	Imported	2019	Dry	High	no	Imported
**PO61**	G3	G1	G2	7	Adult	38	M	Colombia	Imported	2019	Rainy	High	no	Imported
**KY-001**	NA	NA	G2	NA	Adult	40	F	Carreto	GY	2004	Dry	High	no	Local
**KY-002**	NA	NA	G2	NA	Infant	6	M	Carreto	GY	2004	Dry	High	no	Local
**KY-003**	NA	NA	G2	NA	Adult	39	F	Carreto	GY	2004	Dry	High	no	Local
**KY-004**	G1	G1	G2	3	Adult	36	M	Carreto	GY	2004	Dry	High	no	Local
**KY-007**	NA	NA	G2	NA	Adult	19	F	Carreto	GY	2004	Dry	High	no	Local
**KY-008**	G1	G1	G2	3	Infant	11	M	Carreto	GY	2004	Dry	High	no	Local
**KY-010**	NA	NA	G2	NA	Infant	15	M	Carreto	GY	2004	Dry	High	yes	Local

**KY-011**	NA	NA	G2	NA	Child	5	F	Sasardi Mulatupo	GY	2004	Dry	High	no	Local
**KY-016**	G1	G1	G2	3	Adult	53	M	Carreto	GY	2004	Dry	High	no	Local
**KY-017**	G1	G1	G2	3	Infant	13	M	Carreto	GY	2004	Dry	High	no	Local
**KY-018**	G1	G1	G2	3	Adult	34	F	Carreto	GY	2004	Dry	High	no	Local
**KY-022**	NA	NA	G2	NA	Adult	23	F	Sasardi Mulatupo	GY	2004	Dry	High	no	Local
**KY-025**	NA	NA	G2	NA	Adult	26	F	Carreto	GY	2004	Dry	High	yes	Local
**KY-030**	NA	NA	G2	NA	Infant	14	M	Ogosucur	GY	2004	Dry	NA	no	Local
**KY-031**	NA	NA	G2	NA	Adult	25	F	Carreto	GY	2004	Dry	Medium	yes	Local
**KY-032**	G1	G1	G2	3	Adult	28	M	Carreto	GY	2004	Dry	Medium	no	Local
**KY-037**	G1	G1	G2	3	Adult	47	M	Sasardi Muluatupo	GY	2004	Dry	High	no	Local
**KY-038**	NA	NA	G2	NA	Adult	40	M	Sasardi Muluatupo	GY	2004	Dry	High	no	Local
**KY-042**	NA	NA	G2	NA	Adult	33	M	Playon Chico	GY	2004	Dry	High	no	Local
**KY-044**	G2	G1	NA	NA	Child	4	F	Irgandi	GY	2004	Rainy	High	no	Local
**KY-049**	NA	NA	G2	NA	Adult	32	M	Ustupo	GY	2004	Dry	NA	no	Local
**KY-053**	NA	NA	G2	NA	Adult	27	F	Carreto	GY	2004	Dry	High	yes	Local
**KY-056**	G1	G1	G2	3	Adult	33	F	Irgandi	GY	2004	Dry	Medium	no	Local
**KY-057**	G1	G1	G3	9	Adult	31	M	Playon Grande	GY	2004	Dry	NA	no	Local
**KY-063**	NA	NA	G2	NA	Adult	32	M	Playon Grande	GY	2004	Dry	High	no	Local
**KY-065**	G1	G1	G2	3	Adult	28	M	Muluatupo	GY	2004	Dry	High	no	Local
**KY-066**	G1	G1	G2	3	Adult	78	M	Muluatupo	GY	2004	Dry	High	yes	Local
**KY-069**	G1	G1	G2	3	Adult	18	F	Navagandi	GY	2004	Dry	High	no	Local
**KY-070**	G1	G1	G2	3	Adult	35	M	Sasard muluatupo	GY	2004	Dry	High	no	Local
**KY-073**	NA	NA	G2	10	Adult	30	F	Ogobsucum	GY	2004	Dry	NA	no	Local
**KY-075**	NA	NA	G2	NA	Adult	49	F	Ustupo	GY	2004	Dry	High	no	Local
**KY-076**	G1	G1	G2	3	Adult	30	F	Ogobsucum	GY	2004	Dry	NA	no	Local
**KY-079**	G1	G1	G2	3	Child	4	M	Navagandi	GY	2004	Dry	NA	no	Local
**KY-082**	NA	NA	G2	NA	Adult	56	M	Isla Cuba	GY	2004	Dry	NA	no	Local
**KY-085**	NA	NA	G2	NA	Infant	15	F	Navagandi	GY	2004	Dry	High	yes	Local
**KY-086**	G1	G1	G2	3	Infant	9	F	Navagandi	GY	2004	Dry	High	no	Local
**KY-087**	G1	G1	G2	3	Adult	28	F	Navagandi	GY	2004	Dry	High	no	Local
**KY-088**	G1	G1	G2	3	Adult	35	M	Navagandi	GY	2004	Dry	Medium	yes	Local
**KY-089**	G1	G1	G2	3	Adult	35	F	Navagandi	GY	2004	Dry	High	yes	Local
**KY-092**	G1	G1	G2	3	Adult	41	M	La miel	GY	2004	Dry	NA	no	Local
**KY-093**	NA	NA	G2	NA	Child	4	M	La miel	GY	2004	Dry	NA	no	Local
**KY-094**	NA	NA	G2	NA	Infant	8	F	Armila	GY	2004	Dry	NA	no	Local
**KY-095**	NA	NA	G2	NA	Infant	8	M	Armila	GY	2004	Rainy	NA	no	Local
**KY-096**	NA	NA	G2	NA	Infant	9	M	Armila	GY	2004	Dry	NA	no	Local
**KY-097**	G1	G1	G2	3	Adult	46	F	Navagandi	GY	2004	Dry	High	yes	Local

**KY-098**	G1	G1	G2	3	Adult	26	M	Sasardi	GY	2004	Rainy	High	yes	Local
**KY-105**	G1	G1	G2	3	Infant	15	F	Nueva Anachucuna	GY	2004	Rainy	High	yes	Local
**KY-107**	G1	G1	G2	3	Adult	20	F	Carreto	GY	2004	Dry	High	no	Local
**KY-108**	G1	G1	G2	3	Adult	37	M	Cuba	GY	2004	Dry	High	no	Local
**KY-114**	G1	G1	G2	3	Adult	38	F	Isla Pino	GY	2004	Dry	Low	no	Local
**KY-128**	NA	NA	G2	NA	Infant	14	M	Manduyala	GY	2004	Dry	NA	no	Local
**KY-129**	G1	G1	G2	3	Adult	19	M	Nargana	GY	2004	Rainy	Low	no	Local
**KY-148**	G4	G1	NA	NA	Adult	39	M	Puerto Obaldia	GY	2014	Rainy	Low	no	Local
**KY-152**	G2	G1	NA	NA	Adult	51	M	Nargana	GY	2015	Rainy	NA	no	Local

G [Genotype] (1 to 3 and NA for not analyzed); H [Haplotype] (1 to 12 and NA for not analyzed); A [Age] (Child, Infant, Adult); Sex (M: Male, F: Female); Province (D: Darien, PE: Panamá Este, GY: Guna Yala, Imported); CY [Collection Year]; S [Season] (Rainy, Dry); PL [Parasite Load] (High, Medium, Low and NA for not analyzed); GP[Gametocyte Presence] (Yes or No); [Origin] (Local or imported based on patients´ travel history.

Publisher's Note: MDPI stays neutral with regard to jurisdictional claims in published maps and institutional affiliations.

© 2020 by the authors. Licensee MDPI, Basel, Switzerland. This article is an open access article distributed under the terms and conditions of the Creative Commons Attribution (CC BY) license (http://creativecommons.org/licenses/by/4.0/).
